# Gender differences in the relationship between depressive symptoms and diabetes associated with cognitive-affective symptoms

**DOI:** 10.1192/bjo.2024.764

**Published:** 2024-11-05

**Authors:** Shakila Meshkat, Vanessa K. Tassone, Sarah Dunnett, Hilary Pang, Michelle Wu, Josheil K. Boparai, Hyejung Jung, Wendy Lou, Venkat Bhat

**Affiliations:** Interventional Psychiatry Program, St. Michael's Hospital, Toronto, Canada; Department of Psychiatry, University of Toronto, Toronto, Canada; Department of Biostatistics, Dalla Lana School of Public Health, University of Toronto, Toronto, Canada; Institute of Medical Science, Temerty Faculty of Medicine, University of Toronto, Toronto, Canada; Mental Health and Addictions Services, St. Michael's Hospital, Toronto, Canada

**Keywords:** Depressive disorder, diabetes mellitus, prediabetic state, gender differences, NHANES

## Abstract

**Background:**

Despite the frequent co-occurrence of depression and diabetes, gender differences in their relationship remain unclear.

**Aims:**

This exploratory study examined if gender modifies the association between depressive symptoms, prediabetes and diabetes with cognitive-affective and somatic depressive symptom clusters.

**Method:**

Cross-sectional analyses were conducted on 29 619 participants from the 2007–2018 National Health and Nutrition Examination Survey. Depressive symptoms were measured by the nine-item Patient Health Questionnaire. Multiple logistic regression was used to analyse the relationship between depressive symptoms and diabetes. Multiple linear regression was used to analyse the relationship between depressive symptom clusters and diabetes.

**Results:**

The odds of having depressive symptoms were greater in those with diabetes compared to those without. Similarly, total symptom cluster scores were higher in participants with diabetes. Statistically significant diabetes–gender interactions were found in the cognitive-affective symptom cluster model. Mean cognitive-affective symptom scores were higher for females with diabetes (coefficient = 0.23, CI: 0.10, 0.36, *P* = 0.001) than males with diabetes (coefficient = −0.05, CI: −0.16, 0.07, *P* = 0.434) when compared to the non-diabetic groups.

**Conclusions:**

Diabetes was associated with higher cognitive-affective symptom scores in females than in males. Future studies should examine gender differences in causal pathways and how diabetic states interact with gender and influence symptom profiles.

Depression and diabetes are significant contributors to disability globally^1^ and are frequently comorbid, with 28% of individuals with diabetes also suffering from a depressive disorder.^2^ When both conditions are present, patients report significantly greater functional impairment than those with either depression or diabetes alone.^3^ The presence of depression in diabetes can reduce treatment adherence and increase complications and risk of death.^4^ Individuals with prediabetes can reverse their condition through lifestyle modifications.^5^ However, when co-occurring with depression, these changes may be more difficult to implement,^6^ and the two conditions synergistically interact to increase the risk of diabetes.^7^

There are significant gender differences in the development, psychological impact and management of diabetes.^8,9^ As such, it is essential to examine the relationship between diabetes and depressive symptoms with this in mind. While the prevalence of depression is higher among females with diabetes as compared to males,^10^ meta-analyses that stratify results by gender and include predominantly longitudinal analyses have found greater associations between depression and diabetes in males than in females.^10,11^ Other research has shown a relationship between depression and diabetes among females but not males,^12,13^ or significantly greater odds of depression for females versus males.^14^ Moreover, there is evidence for relationships between depression and both insulin resistance^15^ and prediabetes^16^ in both males and females, though gender-based differences are not clear.

Considering depression as a combination of two symptom clusters (i.e. somatic and cognitive-affective) rather than one uniform condition may aid in understanding how the relationship with diabetes differs by gender. Based on the *Diagnostic and Statistical Manual of Mental Disorders, Fifth Edition*^17^ (DSM) criteria, somatic symptoms relate to sleep, energy, appetite and psychomotor slowing/restlessness, whereas symptoms relating to anhedonia, mood, guilt, concentration and suicidal ideation are cognitive-affective. Analyses that consider such symptom clusters have been shown to provide significant benefits over those that examine total depression only,^18^ particularly when investigating health outcomes.^19^ The cognitive-affective symptom cluster has been shown to be associated with an individual's perception of their unmet psychological care needs, while somatic symptoms are not.^20^ Without distinguishing by gender, research has found diabetes,^21^ insulin resistance^22^ and metabolic syndrome (MetS)^23^ – which are correlated with diabetes^24^ – to be primarily associated with somatic symptoms of depression. Only one study has examined depression symptom clusters by gender, finding that associations were primarily driven by somatic symptoms in both males and females.^23^ However, that study examined MetS and not diabetes or prediabetes. Further, despite research suggesting that the association between diabetes and depression differs according to gender,^10,11^ no study has examined how depressive symptom clusters may differ by gender in people with diabetes or prediabetes.

To fill these knowledge gaps, this exploratory study examined the associations of depressive symptoms and symptom cluster scores with diabetes status, including gender-based interactions. We hypothesised that (a) individuals with known prediabetes and diabetes would have statistically significantly higher odds of having depressive symptoms than non-diabetic individuals, and that this would occur to a greater extent in females than in males, (b) somatic symptom scores would be statistically significantly higher in prediabetic and diabetic individuals compared to non-diabetic individuals, with no gender differences, and (c) cognitive-affective symptom scores would not be statistically significantly higher in prediabetic and diabetic individuals compared to non-diabetic individuals, but that scores would be statistically significantly higher in females with diabetes and prediabetes than in males.

## Method

### Study population

This study used data from the 2007–2018 cycles of the National Health and Nutrition Examination Survey (NHANES). NHANES is an annual cross-sectional survey administered by the National Center for Health Statistics (NCHS), part of the Centers for Disease Control and Prevention (CDC). Data were collected from a sample of the non-institutionalised US population. The NHANES data collection protocols are approved each year by the NCHS Ethics Review Board and informed consent is obtained from all participants. More information on the protocols and sampling methods is available on the CDC website (https://www..cdc.gov/nchs/nhanes/analyticguidelines.aspx#). The study sample included males and females aged 20+ years who completed the Mental Health – Depression Screener (items DPQ010 to DPQ090) and the Diabetes Questionnaire (items DIQ010 and/or DIQ160). While NHANES does not differentiate between the type of diabetes, participants who met all of the following criteria were excluded, as type 1 diabetes was likely: (a) diagnosed with diabetes prior to age 30 (DID040), (b) started insulin therapy within 1 year of diabetes diagnosis (i.e. age minus length of time taking insulin [DID060] = within 1 year of age first diagnosed with diabetes [DID040]) and (c) taking insulin when surveyed (DIQ050).^25^

### Exposure variable

Diabetes status was categorised as (a) no diabetes, (b) known prediabetes or (c) diabetes, assessed through self-report via the questions DIQ010, ‘Other than during pregnancy, have you ever been told by a doctor or health professional that you have diabetes or sugar diabetes?’ and DIQ160, ‘Have you ever been told by a doctor or other health professional that you have any of the following: prediabetes, impaired fasting glucose, impaired glucose tolerance, borderline diabetes or that your blood sugar is higher than normal but not high enough to be called diabetes or sugar diabetes?’ Individuals responding ‘yes’ to DIQ010 were categorised as having diabetes. Those who responded ‘no’ to DIQ010 were asked DIQ160. Individuals responding ‘borderline’ to DIQ010 or ‘yes’ to DIQ160 were categorised as having known prediabetes. Individuals responding ‘no’ to DIQ160 were categorised as non-diabetic.

### Outcome variable

The presence of depressive symptoms was measured using the Mental Health – Depression Screener, which asks participants to complete the Patient Health Questionnaire-9 (PHQ-9). The PHQ-9 comprises nine items, which assess the frequency of depressive symptoms experienced over the past 2 weeks, based on diagnostic criteria for major depressive disorder (MDD) from the *DSM – Fourth Edition.*^26^ The items on this scale are scored from 0 (experienced no days) to 3 (experienced nearly every day). Answers to items 1–9 on the PHQ-9 were summed and participants were categorised as having depressive symptoms (score ≥ 10) or not (score < 10). Symptom clusters were determined by summing scores to questions within each cluster (questions 1, 2, 6, 7 and 9 for cognitive-affective and questions 3, 4, 5 and 8 for somatic) and were kept as continuous values. Symptom clusters were defined in this way to align with existing diabetes/prediabetes and depression-related studies.^26,27^

### Statistical analysis

Statistical analyses were performed using R, version 4.2.1 for MacOS, and the package ‘survey’ to account for survey weights. Mobile exam centre survey weights were divided by six to account for the merging of six survey cycles. Categorical variables were described as raw frequency and weighted percent in the study population demographic characteristics table, while continuous variables were described as weighted mean and s.d. A chi-square test of independence was used to check for statistically significant (*P*-value ≤0.05) differences in categorical demographic characteristics among the gender-dependent diabetes groups, and a *t*-test was used to test for differences in continuous variables. Multiple logistic regression was conducted to assess the relationship between diabetes status and presence of depressive symptoms (yes/no). A sensitivity analysis with depressive symptoms as a continuous measure, rather than categorical, was run using a multiple linear regression model. Multiple linear regression models were also used to assess the relationship between diabetes status and cognitive-affective or somatic symptom cluster scores. An additional sensitivity analysis was run with item 8 (psychomotor retardation) included in the cognitive-affective symptom cluster rather than the somatic symptom cluster.^27^ Next, additional models were run to test the interaction effects between diabetes status and gender for all previous models. Where interaction terms were statistically significant, a subgroup analysis by gender was conducted to further investigate the relationship between presence of depressive symptoms or symptom cluster scores and diabetes status. Statistical significance was set to *P* < 0.01 to account for multiple testing and reduce Type 1 error for all models.

Shortlisted covariates based on prior literature included age (continuous by year), gender (female or male), body mass index (BMI; <25 kg/m^2^, ≥25 to <30 kg/m^2^, ≥30 kg/m^2^), race (non-Mexican White, non-Mexican Black, Mexican Hispanic, other Hispanic, other [including multi-racial]), poverty-income ratio (PIR; low income ≤1.3, mid-to-high income >1.3),^28^ and sedentary activity (continuous by minutes per week). Backward stepwise selection was used to validate which covariates would be included in the multiple regression models with a cutoff of *P* < 0.10. All covariates were selected in all models, with the exception of the cognitive-affective model, which did not include sedentary activity. Symptom cluster models also controlled for the opposite symptom cluster.

## Results

### Descriptive statistics

**Fig. 1:**
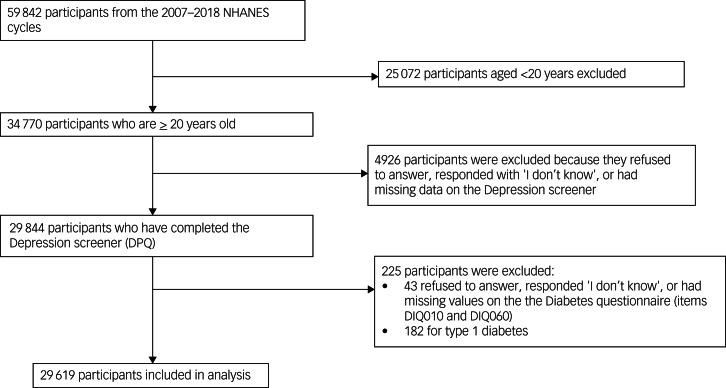
Flowchart of National Health Examination and Nutrition Examination Survey (NHANES) participants included in the final study population.

The study population included 29 619 participants. Figure 1 shows participants’ inclusion into the study. The mean age of participants was 47.64 years (s.d. = 17) and 15 052 (51.32%) were female. Moreover, 23 224 (82%) participants were non-diabetic, 2548 (8.52%) were prediabetic and 3847 (9.48%) were diabetic (Table 1, Supplementary Table 1 available at https://doi.org/10.1192/bjo.2024.764). The overall prevalence of depressive symptoms in this sample was 8.03%, with a prevalence of 10.12 and 5.82% in females and males, respectively. Across the total sample, mean cognitive-affective symptom scores were 1.20 (s.d. = 2.21) and mean somatic symptom scores were 1.89 (s.d. = 2.31). Respective female and male mean scores were 1.37 (s.d. = 2.35) and 1.02 (s.d. = 2.05) for cognitive-affective symptoms, and 2.19 (s.d. = 2.44) and 1.57 (s.d. = 2.12) for somatic symptoms.

**Table 1:** Demographics of study sample, based on diabetes status and gender

	No diabetes			Prediabetes			Diabetes		
	Females	Males	*P*-value	Females	Males	*P*-value	Females	Males	*P*-value
Sample size	11 773	11 451		1428	1120		1851	1996	
Age, years (mean, s.d.)	46.44 (17.06)	44.32 (16.32)	<0.001	53.81 (15.56)	54.70 (14.16)	0.226	60.76 (13.31)	61.44 (11.85)	0.197
Depressive symptoms – yes	1201 (8.92)	668 (5.29)	<0.001	186 (13.09)	119 (8.84)	0.020	337 (17.94)	199 (7.93)	<0.001
PHQ-9 score (mean, s.d.)	3.32 (4.19)	2.47 (3.64)	<0.001	4.26 (4.62)	3.28 (4.48)	<0.001	5.04 (5.25)	3.04 (4.15)	<0.001
Cognitive-affective symptom scores (mean, s.d.)	1.26 (2.24)	0.98 (1.97)	<0.001	1.64 (2.56)	1.33 (2.50)	0.034	2.06 (2.86)	1.15 (2.24)	<0.001
Somatic symptom scores (mean, s.d.)	2.06 (2.35)	1.49 (2.05)	<0.001	2.61 (2.53)	1.95 (2.43)	<0.001	2.98 (2.88)	1.89 (2.33)	<0.001
Race			<0.001			0.073			0.002
Non-Hispanic White	5061 (68.37)	4930 (66.85)		524 (65.43)	480 (70.21)		588 (58.77)	738 (64.17)	
Non-Hispanic Black	2390 (11.09)	2346 (10.10)		325 (11.93)	242 (9.32)		529 (17.49)	508 (12.77)	
Mexican Hispanic	1715 (7.56)	1694 (9.39)		216 (7.73)	154 (7.92)		334 (9.32)	331 (8.89)	
Other Hispanic	1292 (5.73)	1113 (5.95)		164 (5.92)	98 (4.99)		220 (5.87)	196 (5.17)	
Other race – including multiracial	1315 (7.25)	1368 (7.70)		199 (9.00)	146 (7.56)		180 (8.56)	223 (9.01)	
Poverty-income ratio			<0.001			0.020			<0.001
Mid-to-high income (>1.3)	7212 (77.36)	7271 (79.72)		900 (79.99)	759 (84.25)		965 (69.38)	1235 (78.86)	
Body mass index			<0.001			0.004			<0.001
<25 kg/m^2^	3940 (36.72)	3430 (28.33)		234 (16.56)	200 (14.72)		207 (9.91)	270 (11.47)	
≥25 to <30 kg/m^2^	3443 (29.26)	4369 (38.71)		363 (27.71)	408 (37.20)		414 (20.13)	662 (30.18)	
≥30 kg/m^2^	4294 (34.03)	3553 (32.96)		825 (55.73)	502 (48.08)		1197 (69.95)	1024 (58.35)	
Minutes of sedentary activity/week (mean, s.d.)	366.39 (198.50)	367.89 (203.69)	0.681	391.15 (205.56)	398.01 (209.32)	0.617	387.48 (212.20)	407.99 (212.13)	0.025

*P*-values < 0.05 denote statistically significant differences between genders, within each diabetes status group (i.e. no diabetes, prediabetes, diabetes). For statistically significant differences across diabetes status groups, see Supplementary Table 1. Categorical variables presented as unweighted frequencies and weighted percentages. Continuous variables presented as weighted mean and s.d.

PHQ-9, Patient Health Questionnaire-9.

### Depressive symptoms

Individuals with known prediabetes (adjusted odds ratio (aOR): 1.63, 95% CI: 1.30, 2.04, *P* < 0.001) or diabetes (aOR: 1.85, CI: 1.58, 2.17, *P* < 0.001) had statistically significantly higher odds of having depressive symptoms compared to those without diabetes (Table 2, Supplementary Table 2). There was no interaction effect between diabetes status and gender for prediabetes (Table 3, Supplementary Table 3). The interaction between diabetes status and gender was not statistically significant for diabetes in the main analysis based on our threshold for statistical significance (*P =* 0.036) (Table 3). However, in the sensitivity analysis wherein depressive symptoms were treated continuously, this interaction was statistically significant (Supplementary Table 3). Females with diabetes had an average total depressive symptom score that was 1.44 points greater than females without diabetes (adjusted coefficient (aCoeff.) = 1.44, CI: 1.10, 1.78, *P* < 0.001), whereas males with diabetes showed a smaller difference in mean scores than their non-diabetic counterparts (aCoeff. = 0.71, CI: 0.44, 0.98, *P* < 0.001) (Supplementary Table 4). As such, subsequent subgroup analyses by gender were conducted for the main analysis based on the overall trend of results and its potential clinical relevance in suggesting that the association between diabetes status and depressive symptoms may differ by gender. In the main analysis, the odds of depressive symptoms for females with diabetes were more than two times the odds of depressive symptoms for females without diabetes (aOR: 2.05, CI: 1.65, 2.54, *P* < 0.001), whereas the odds of depressive symptoms for males with diabetes was 52% higher than males without diabetes (aOR: 1.52, CI: 1.19, 1.93, *P* = 0.001) (Table 4, Supplementary Table 4).

**Table 2:** Results of main effect multiple logistic and linear regressions

Exposure	Depressive symptoms		Cognitive-affective symptom cluster		Somatic symptom cluster	
	aOR (95% CI)	*P*-value	aCoeff. estimate (95% CI)	*P*-value	aCoeff. estimate (95% CI)	*P*-value
Diabetes status						
No diabetes	1 (ref)	–	0 (ref)	–	0 (ref)	–
Prediabetes	1.63 (1.30, 2.04)	**<0.001**	0.07 (−0.07, 0.22)	0.315	0.24 (0.13, 0.35)	**<0.001**
Diabetes	1.85 (1.58, 2.17)	**<0.001**	0.09 (0.00, 0.18)	0.039	0.30 (0.19, 0.41)	**<0.001**

aOR, adjusted odds ratio; aCoeff., adjusted coefficient; ref, reference. *P*-values < 0.01 (shown in bold) denote statistical significance. Depressive symptom and somatic symptom cluster models adjusted for age, gender, body mass index, race, poverty-income ratio, sedentary activity; cognitive-affective symptom cluster model adjusted for the same variables with the exception of sedentary activity; somatic and cognitive-affective symptom cluster models additionally controlled for the opposite symptom cluster.

**Table 3:** Results of multiple logistic and linear regressions with interaction effects

Exposure	Depressive symptoms		Cognitive-affective symptom cluster		Somatic symptom cluster	
	aOR (95% CI)	*P*-value	aCoeff. estimate (95% CI)	*P*-value	aCoeff. estimate (95% CI)	*P*-value
Diabetes status						
No diabetes	1 (ref)	–	0 (ref)	–	0 (ref)	–
Prediabetes	1.55 (1.16, 2.07)	**0.004**	0.06 (−0.13, 0.25)	0.515	0.26 (0.10, 0.42)	**0.001**
Diabetes	2.08 (1.68, 2.57)	**<0.001**	0.25 (0.12, 0.39)	**<0.001**	0.31 (0.16, 0.46)	**<0.001**
Gender interaction						
Prediabetes × male	1.16 (0.78, 1.72)	0.449	0.03 (−0.23, 0.29)	0.824	−0.05 (−0.30, 0.20)	0.682
Diabetes × male	0.73 (0.55, 0.98)	0.036	−0.31 (−0.49, −0.13)	**0.001**	−0.02 (−0.19, 0.15)	0.832

aOR, adjusted odds ratio; aCoeff., adjusted coefficient; ref, reference. *P*-values < 0.01 (shown in bold) denote statistical significance. Depressive symptom and somatic symptom cluster models adjusted for age, body mass index, race, poverty-income ratio, sedentary activity; cognitive-affective symptom cluster model adjusted for the same variables with the exception of sedentary activity; somatic and cognitive-affective symptom cluster models additionally controlled for the opposite symptom cluster.

**Table 4:** Subgroup models following statistically significant interaction between diabetes status and gender

Depressive symptoms				
	Female		Male	
	aOR (95% CI)	*P*-value	aOR (95% CI)	*P*-value
No diabetes	1 (ref)	–	1 (ref)	*–*
Prediabetes	1.50 (1.13, 2.01)	**0.006**	1.83 (1.34, 2.50)	**<0.001**
Diabetes	2.05 (1.65, 2.54)	**<0.001**	1.52 (1.19, 1.93)	**0.001**

Cognitive-affective symptom cluster				
	Female		Male	
	aCoeff. estimate (95% CI)	*P*-value	aCoeff. estimate (95% CI)	*P*-value
No diabetes	0 (ref)	–	0 (ref)	–
Prediabetes	0.05 (−0.14, 0.24)	0.601	0.10 (−0.10, 0.30)	0.334
Diabetes	0.23 (0.10, 0.36)	**0.001**	−0.05 (−0.16, 0.07)	0.434

Note: aOR, adjusted odds ratio; aCoeff., adjusted coefficient; ref, reference. *P*-values < 0.01 (shown in bold) denote statistical significance. Depressive symptom model adjusted for age, body mass index (BMI), race, povery-income ratio (PIR), sedentary activity; cognitive-affective symptom model adjusted for age, BMI, race, PIR, somatic symptom cluster.

### Cognitive-affective symptom cluster score

Based on our threshold for statistical significance, neither individuals with diabetes (aCoeff. = 0.09, CI: 0.00, 0.18, *P* = 0.039) or known prediabetes (aCoeff. = 0.07, CI: −0.07, 0.22, *P* = 0.315) had statistically significantly higher mean cognitive-affective symptom cluster scores than non-diabetic individuals in the main analysis; this became significant for diabetes in the sensitivity analysis wherein psychomotor retardation was included in the cognitive-affective symptom cluster (Table 2, Supplementary Table 2). The interaction between diabetes status and gender was statistically significant for diabetes (*P* = 0.001), but not known prediabetes (Table 3), which was consistent in the sensitivity analysis with psychomotor retardation included in the cognitive-affective cluster (Supplementary Table 3). In adjusted subgroup analyses, females with diabetes had a mean cognitive-affective score that was statistically significant, at 0.23 points higher compared to females without diabetes (CI: 0.10, 0.36, *P* = 0.001), whereas males with diabetes had a mean cognitive-affective score that was 0.05 points lower than non-diabetic males (CI: −0.16, 0.07, *P* = 0.434). The association in males was statistically insignificant (Table 4, Supplementary Table 4).

### Somatic symptom cluster score

Individuals with known prediabetes (aCoeff. = 0.24, CI: 0.13, 0.35, *P* < 0.001) and diabetes (aCoeff. = 0.30, CI: 0.19, 0.41, *P* < 0.001) had statistically significantly higher mean somatic symptom cluster scores than non-diabetic individuals (Table 2). Similarly, in the model including an interaction term between gender and known prediabetes or diabetes, the mean somatic symptom scores were statistically significantly higher for the individuals with known prediabetes and diabetes than those with no diabetes; however, these associations did not differ by gender (Table 3). The sensitivity analysis wherein psychomotor retardation was included in the cognitive-affective symptom cluster yielded the same finding (Supplementary Table 3).

## Discussion

Using a population-based sample, the current study investigated the association between diabetes status and depressive symptoms (total overall and subset into clusters) in males and females. Statistically significant interactions between gender and diabetes were found in the total depressive symptom (when treated as a continuous measure) and cognitive-affective symptom analyses, but not in the somatic symptom analysis. Compared to participants without diabetes, females with diabetes had higher mean total depressive symptom scores and cognitive-affective symptom scores than males with diabetes, though cognitive-affective symptom scores were not statistically significantly related to diabetes in males. Gender did not modify the relationship between known prediabetes and depressive symptoms or symptom cluster scores.

Depressive symptoms were statistically significantly greater in females than in males with diabetes, which is in line with previous research.^13,14^ Several mechanisms can explain this finding, including gender dimorphic risk factors that influence both diabetes and depression. For example, depression risk factors such as lower decision latitude and higher job strain,^29^ lower education level^30^ and lower socioeconomic status in childhood^31^ have been found to be associated with diabetes in females than in males.^8^ Sociocultural gender differences in coping with such stressors may be one explanation behind the higher depressive symptom scores found in females compared to males, as it has been suggested that men are more likely to cope by becoming aggressive and participating in activities while women are more likely to ruminate, decrease physical activity and eat more.^12^ Menopause and associated hormone changes also confer a unique risk for both diabetes and depression on females.^12^ Both factors may play a role in gender-based associations between diabetes and depression, as research has found that this relationship differs by age.^13,14,32,33^ In contrast with the results presented in this study, meta-analyses including predominantly longitudinal studies examined depression as a predictor of diabetes and found greater associations between the two conditions in males.^11^ This suggests that heterogeneity in gender-based associations between depressive symptoms and diabetes may in part stem from variations in study design and from which condition is examined as the precipitating factor. This is supported by a longitudinal study that found a greater positive association in females when diabetes predicted the development of depressed mood than when depressed mood predicted the development of diabetes.^34^ Future research should consider how gender dimorphic risk factors play into gender differences in the diabetes–depression relationship, as well as how causal directions in this relationship may differ.

Previous research has suggested that cognitive-affective symptoms of depression, independent of somatic symptoms, are not associated with diabetes^35^ or insulin resistance.^22^ While this was the case for males in our sample, we found that females with diabetes had statistically significantly higher mean cognitive-affective scores than females without diabetes. This may be explained by the psychological impact of the complications or burdens that can accompany a diabetes diagnosis, known as ‘diabetes distress’. Measures of diabetes distress, including feelings of worry, guilt and frustration with the diagnosis,^34^ are more aligned with cognitive-affective symptoms of depression than somatic symptoms. Existing research supports a greater magnitude of this effect in females than in males.^36,37^ Further, the lack of significance for females with known prediabetes in our sample suggests that known prediabetes is not associated with the same cognitive distress, perhaps because the condition can be reversed. The psychological burden of awareness of diabetes may also explain why our results differ from previous research using similar methods. Specifically, Wiltink et al.^35^ found no significant association between cognitive-affective symptoms and diabetes; however, both those with known (identified through self-report) and unknown (identified through blood tests as part of the study) diabetes were included in the diabetic group. In contrast, the present study examined only known diabetes or prediabetes, suggesting that knowledge of diabetes might be a key factor influencing cognitive-affective symptoms of depression.

Somatic depressive symptoms did not differ between males or females with either known prediabetes or diabetes when compared to their non-diabetic counterparts. Research suggests that females with depression may be more likely than males to exhibit somatic symptoms as part of MDD^38,39^ and endorse somatic symptoms at a greater rate.^39^ Our results suggest that the presence of diabetes or known prediabetes does not statistically significantly alter this pattern.

Study findings did not support our hypothesis that the relationship between depressive symptoms and prediabetes would mirror that of diabetes. Results showed a trend in the opposite direction, with males experiencing greater odds of having depressive symptoms and higher mean cognitive-affective scores, though the associations themselves and the interactions did not reach significance. This may be due to differential types of prediabetes experienced across genders, where males more commonly experience combined impaired glucose tolerance and impaired fasting glucose type prediabetes,^40,41^ which is most strongly associated with depression.^16^ However, further research is needed to investigate the role of gender in the relationship between prediabetes and depressive symptoms and how this relationship may differ from that of diabetes.

This study is not without limitations. While the nationally representative sample allows for the inclusion of individuals who are not seeking care for diabetes or depressive symptoms, the self-report of diabetes status limits diabetes and prediabetes groups to those who are aware of their condition. However, the awareness of diabetes status is associated with depression regardless of metabolic status, and self-report measures of diabetes have been shown to have high sensitivity and specificity.^42^ Moreover, the measurement of depressive symptoms was based solely on total PHQ-9 scores. Using a self-report scale, rather than a semi-structured interview or confirmed clinical diagnosis, limits our study to investigating the association of diabetes and prediabetes with presence of depressive symptoms rather than MDD. Several other conditions (e.g. bereavement, adjustment disorder and substance use disorder) may be associated with elevated PHQ-9 scores, and, as such, it is possible that participants who were categorised as having depressive symptoms were experiencing a condition other than MDD. Finally, the use of cross-sectional data limits the conclusions that can be drawn to correlation and presents the possibility of non-response bias. This study is also limited by the lack of comorbidity analysis.

Results from this study suggest that diabetes is associated with higher total depressive symptom scores and cognitive-affective symptom scores in females. Future studies should examine causal pathways in the diabetes-depressive symptom relationship, while also considering gender to determine how differing trajectories explain the heterogeneity of research in this area. Further, studies should examine gender differences in the distinction between diabetes distress and depressive symptoms. Studies that examine depression symptom profiles and diabetes status should consider how results may differ across genders, as well as how prediabetic or diabetic state and diagnosed or undiagnosed diabetes influence these symptom profiles.

## Supporting information

Meshkat et al. supplementary material Meshkat et al. supplementary material

## Data Availability

The data that support the findings of this study are available from the corresponding author, V.B., upon reasonable request.
