# Supplementary material for: Gender differences in the relationship between depressive symptoms and diabetes associated with cognitive-affective symptoms
Source: BJPsych Open. 2024 Nov 5;10(6):e192. doi: 10.1192/bjo.2024.764 (PMC11698216; doi:10.1192/bjo.2024.764)
Supplement: Meshkat et al. supplementary material [file S2056472424007646sup001.docx]

Supplementary Material

**Table S1.** Demographics by diabetes status, regardless of sex.

|  | No Diabetes | Prediabetes | Diabetes | *p-value* |
| --- | --- | --- | --- | --- |
| Sample size | 23224 | 2548 | 3847 |  |
| Sex - female | 11773 (51.16) | 1428 (55.61) | 1851 (48.85) | 0.001 |
| Age (mean, SD) | 45.40 (16.74) | 54.21 (14.96) | 61.11 (12.59) | <0.001 |
| Depressive symptoms - yes | 1869 (7.15) | 305 (11.20) | 536 (12.82) | <0.001 |
| PHQ-9 (mean, SD) | 2.90 (3.95) | 3.82 (4.58) | 4.02 (4.82) | <0.001 |
| Cognitive-affective symptom scores (mean, SD) | 1.12 (2.12) | 1.50 (2.54) | 1.60 (2.60) | <0.001 |
| Somatic symptom scores (mean, SD) | 1.78 (2.23) | 2.32 (2.51) | 2.42 (2.67) | <0.001 |
| Race |  |  |  | <0.001 |
| Non-Hispanic White | 9991 (67.63) | 1004 (67.55) | 1326 (61.53) |  |
| Non-Hispanic Black | 4736 (10.61) | 567 (10.77) | 1037 (15.07) |  |
| Mexican Hispanic | 3409 (8.46) | 370 (7.82) | 665 (9.10) |  |
| Other Hispanic | 2405 (5.84) | 262 (5.51) | 416 (5.51) |  |
| Other race - including multiracial | 2683 (7.47) | 345 (8.36) | 403 (8.79) |  |
| Poverty-income ratio |  |  |  | <0.001 |
| High income (>1.3) | 14483 (78.51) | 1659 (81.87) | 2200 (74.23) |  |
| Body mass index |  |  |  | <0.001 |
| <25 kg/m^2^ | 7370 (32.62) | 434 (15.75) | 477 (10.71) |  |
| ≥25 to <30 kg/m^2^ | 7812 (33.87) | 771 (31.91) | 1076 (25.26) |  |
| ≥30 kg/m^2^ | 7847 (33.51) | 1327 (52.34) | 2221 (64.03) |  |
| Minutes of sedentary activity/week (mean, SD) | 367.12 (201.05) | 394.20 (207.23) | 398.00 (212.38) | <0.001 |

Note: *p*-values <0.05 denote statistically significant differences across. Categorical variables presented as unweighted frequencies and weighted percentages. Continuous variables presented as weighted means and standard deviations.

**Table S2.** Results of main effect multiple logistic and linear regressions for sensitivity analyses (i.e., depressive symptoms as continuous, PHQ-9 item 8 classified as cognitive-affective rather than somatic).

| Exposure | Total Depressive Symptom Score | | Cognitive-Affective Symptom Cluster | | Somatic Symptom Cluster | |
| --- | --- | --- | --- | --- | --- | --- |
|  | aCoeff. Estm. (95% CI) | *p-value* | aCoeff. Estm. (95% CI) | *p-value* | aCoeff. Estm. (95% CI) | *p-value* |
| Diabetes Status |  |  |  |  |  |  |
| No Diabetes | 0 (ref) | — | 0 (ref) | — | 0 (ref) | — |
| Prediabetes | 0.87 (0.58, 1.16) | **< 0.001** | 0.09 (-0.07, 0.25) | 0.254 | 0.23 (0.13, 0.33) | **< 0.001** |
| Diabetes | 1.08 (0.85, 1.31) | **< 0.001** | 0.16 (0.05, 0.27) | **0.007** | 0.27 (0.16, 0.38) | **< 0.001** |

Note: aCoeff. Estm. = adjusted coefficient estimate, *p*-values <0.01 denote statistical significance. Total depressive symptom score and somatic symptom cluster models adjusted for age, sex, BMI, race, PIR, sedentary activity; cognitive-affective symptom cluster model adjusted for the same variables with the exception of sedentary activity; somatic and cognitive-affective symptom cluster models additionally controlled for the opposite symptom cluster.

**Table S3.** Results of multiple logistic and linear regressions with interaction effects for sensitivity analyses (i.e., depressive symptoms as continuous, PHQ-9 item 8 classified as cognitive-affective rather than somatic).

| Exposure | Total Depressive Symptom Score | | Cognitive-Affective Symptom Cluster | | Somatic Symptom Cluster | |
| --- | --- | --- | --- | --- | --- | --- |
|  | aCoeff. Estm. (95% CI) | *p-value* | aCoeff. Estm. (95% CI) | *p-value* | aCoeff. Estm. (95% CI) | *p-value* |
| Diabetes Status |  |  |  |  |  |  |
| No Diabetes | 0 (ref) | — | 0 (ref) | — | 0 (ref) | — |
| Prediabetes | 0.88 (0.50, 1.25) | **< 0.001** | 0.07 (-0.15, 0.29) | 0.524 | 0.26 (0.11, 0.40) | **0.001** |
| Diabetes | 1.55 (1.21, 1.89) | **< 0.001** | 0.35 (0.18, 0.51) | **< 0.001** | 0.26 (0.10, 0.42) | **0.002** |
| Sex Interaction |  |  |  |  |  |  |
| Prediabetes × Male | -0.01 (-0.49, 0.47) | 0.965 | 0.06 (-0.22, 0.33) | 0.680 | -0.07 (-0.26, 0.13) | 0.516 |
| Diabetes × Male | -0.92 (-1.31, -0.54) | **< 0.001** | -0.37 (-0.58, -0.15) | **0.001** | -0.01 (-0.18, 0.16) | 0.862 |

Note: aCoeff. Estm. = adjusted coefficient estimate, *p*-values <0.01 denote statistical significance. Total depressive symptom score and somatic symptom cluster models adjusted for age, BMI, race, PIR, sedentary activity; cognitive-affective symptom cluster model adjusted for the same variables with the exception of sedentary activity; somatic and cognitive-affective symptom cluster models additionally controlled for the opposite symptom cluster.

**Table S4.** Subgroup models explaining statistically significant interaction effects between diabetes status and sex for sensitivity analyses (i.e., depressive symptoms as continuous, PHQ-9 item 8 classified as cognitive-affective rather than somatic).

| Total Depressive Symptom Score | | | | |
| --- | --- | --- | --- | --- |
|  | Female | | Male | |
|  | aCoeff. Estm. (95% CI) | *p-value* | aCoeff. Estm. (95% CI) | *p-value* |
| No Diabetes | 0 (ref) | — | 0 (ref) | *—* |
| Prediabetes | 0.81 (0.42, 1.19) | **< 0.001** | 0.91 (0.53, 1.28) | **< 0.001** |
| Diabetes | 1.44 (1.10, 1.78) | **< 0.001** | 0.71 (0.44, 0.98) | **< 0.001** |
| Cognitive-Affective Symptom Cluster | | | | |
|  | Female | | Male | |
|  | aCoeff. Estm. (95% CI) | *p-value* | aCoeff. Estm. (95% CI) | *p-value* |
| No Diabetes | 0 (ref) | — | 0 (ref) | — |
| Prediabetes | 0.06 (-0.16, 0.28) | 0.584 | 0.13 (-0.08, 0.34) | 0.212 |
| Diabetes | 0.33 (0.17, 0.50) | **< 0.001** | -0.01 (-0.16, 0.14) | 0.885 |

Note: aCoeff. Estm. = adjusted coefficient estimate, *p*-values <0.01 denote statistical significance. Total depressive symptom score model adjusted for age, BMI, race, PIR, sedentary activity; cognitive-affective symptom model adjusted for age, BMI, race, PIR, somatic symptom cluster.
